# The immune cell landscape of metastatic uveal melanoma correlates with overall survival

**DOI:** 10.1186/s13046-021-01947-1

**Published:** 2021-05-04

**Authors:** Anna Tosi, Rocco Cappellesso, Angelo Paolo Dei Tos, Valentina Rossi, Camillo Aliberti, Jacopo Pigozzo, Alessio Fabozzi, Marta Sbaraglia, Stella Blandamura, Paola Del Bianco, Vanna Chiarion-Sileni, Antonio Rosato

**Affiliations:** 1 Department of Surgery, Oncology and Gastroenterology, University of Padova, Padova, Italy; 2 Pathological Anatomy Unit, Padova University Hospital, Padova, Italy; 3 Department of Medicine (DIMED), Surgical Pathology Unit, University of Padova, Padova, Italy; 4 Immunology and Molecular Oncology Diagnostics, Veneto Institute of Oncology IOV-IRCCS, Via Gattamelata 64, 35128 Padova, Italy; 5 Departement of Diagnostic & Interventive Radiology-Pederzoli Hospital, Peschiera, VR Italy; 6 Melanoma Oncology Unit, Veneto Institute of Oncology IOV-IRCCS, Padova, Italy; 7 Department of Oncology, Veneto Institute of Oncology IOV-IRCCS, Padova, Italy; 8 Clinical Research Unit, Veneto Institute of Oncology IOV-IRCCS, Padova, Italy

**Keywords:** Metastatic uveal melanoma, Tumor microenvironment, Immune cell, Immune contexture, Immune biomarkers

## Abstract

**Background:**

Uveal melanoma (UM) represents the most common primary intra-ocular malignancy in adults. Up to 50% of the patients develop distant metastases within 10 years from diagnosis, with the liver as the most common site. Upon metastatization, life expectancy strongly reduces and immune checkpoint inhibitors that prove effective in cutaneous melanoma do not modify clinical outcome. To date, few studies have focused on deciphering the immunomodulatory features of metastatic UM microenvironment, and there are no prognostic models for clinical use. This highlights the urgent need to understand the delicate interplay between tumor and immune cells acting at the site of metastasis.

**Methods:**

We collected a patient cohort comprising 21 metastatic UM patients. Hepatic and extra-hepatic UM metastasis samples were studied by multiplex immunofluorescence to assess the tumor immune cell composition. Quantitative analyses were performed to correlate immune cell densities with treatment response, metastasis site and patient survival.

**Results:**

Compared to patients with progressive disease, those with controlled disease had a higher intra-tumoral/peritumoral ratio of CD8 + Granzyme B+ cells, higher density of intra-tumoral CD8+ cytotoxic T lymphocytes (CTL) and an increased percentage of UM cells in close proximity to T lymphocytes, reflecting a role of tumor-killing T cells in the disease. In liver metastases (LM), the intra-tumoral densities of CD163+ tumor-associated macrophages (TAM) and of total CD8+ T cells were higher than in extra-hepatic UM metastases, but the percentage of Granzyme B+ CTL was lower. Moreover, LM displayed more UM cells adjacent to both CTL and TAM, and also more T cells in proximity to TAM, all signs of an impaired immune response. The percentage of activated CTL within the tumor represented a prognostic indicator, as patients with a higher intra-tumoral percentage of CD8 + Granzyme B+ cells had the better outcome. A temptative Immunoscore was generated and proved capable to stratify patients with improved survival. Finally, CD4 + FoxP3+ T cells appeared a crucial population for response to immunotherapy.

**Conclusion:**

The results of this study underly the clinical relevance and functional importance of composition and localization of antitumor effector cells for the progression of UM metastasis.

**Supplementary Information:**

The online version contains supplementary material available at 10.1186/s13046-021-01947-1.

## Background

Uveal melanoma (UM) originates from the uveal tract of the eye [1], and differs from the cutaneous melanoma in risk factors, biological behavior, epidemiology, prognostic features, and molecular profiles. Indeed, UM has an extremely low mutational burden contrary to cutaneous melanoma [2], which harbors a high rate of C > T transitions and double CC > TT mutations induced by UV exposure, and hot-spot mutations in *BRAF* or *RAS* or loss of function mutations in *NF1* [3]. On the other hand, the vast majority (85–95%) of metastatic UM harbors monosomy 3, *GNA11* or *GNAQ* mutations [4]. Despite the improvement and effectiveness of local tumor control (80% at 5 years), the high tendency to metastasize has not changed [5] and still up to 50% of the patients develop distant metastases within 10 years after diagnosis. The liver is the most common site (80–90%), followed by lung (29%) and bone (17%) [6, 7]. Once metastases are present, the disease course is generally aggressive and the prognosis remains poor, with a median overall survival (OS) of 13.4 months [8], and a 2-year OS rate of only 8% [9].

Liver metastasis is a relevant aspect of clinical course, and liver failure is almost the exclusive cause of death even when other visceral sites are involved. The median survival of patients who develop UM liver metastasis ranges between 6 to 12 months, as compared to 19–28 months for patients who first metastasize in other sites [6, 10, 11]. In the metastatic stage, UM systemic therapy largely derives from approaches effective against cutaneous melanoma. Additionally, a variety of local liver-directed treatment options have been investigated, including surgical resection, hepatic artery embolization, hepatic arterial chemotherapy infusion, and radiofrequency, but none of them has resulted in an improved survival in metastatic disease [12]. Further, CTLA-4 (ipilimumab) and PD-1 (nivolumab, pembrolizumab) inhibitors as monotherapy in sequence or combined, and targeted therapies with anti-angiogenic and kinase inhibitors [13] have been also tested, but with disappointing results or only marginal success to date [14–16]. Recently, the adoptive transfer of in vitro expanded autologous tumor-infiltrating lymphocytes (TILs) has been reported to mediate objective tumor regression in some patients with metastatic UM, thus fostering further investigation on the role of immune cells in this challenging disease [17].

Assays using a variety of molecular techniques have the ability to analyze the primary tumor to predict prognosis and the risk of metastasis [18, 19]. Differently from what observed in other cancer types [20], evaluation of the prognostic impact of immune system in primary UM has revealed that high densities of tumor-associated macrophages (TAMs) and TILs are associated with a poor prognosis, and a high risk of metastasis [21–23]. However, few studies have focused on deciphering the immunomodulatory features of metastatic UM microenvironment [24, 25], largely due to the difficulty in acquiring specimens that often derive from percutaneous biopsies. Moreover, to date there are no prognostic models for clinical use in newly diagnosed metastatic disease [26, 27]. Circulating UM cells that enter the liver encounter a unique immune system, as liver also acts as an immune-modulating organ devoted to quickly defeat gastrointestinal-derived pathogens, and at the same time to maintain tolerance against harmless food antigens [28]. Therefore, interaction between liver immune system and cancer cells provide a complex tumor microenvironment that could help UM cells to evade an antitumor immune response. Thus, efforts must be put in place to understand the delicate interplay that occurs between tumor and immune cells acting at the site of metastasis, to allow the identification of prognostic/predictive factors which could facilitate the tailored management of patients and improve survival outcomes.

We designed a study aimed at analysing the density and the spatial distribution of immune cell subpopulations in a cohort of patients with hepatic and extra-hepatic metastasis from UM. The final goal was to identify immune biomarkers able to capture the immune contexture of tumor microenvironment that could stratify patients with better prognosis, in order to guide patient care and to facilitate future rational trial design to target appropriate metastatic UM patient subgroups.

## Methods

### Patient characteristics

The study cohort comprised 21 patients diagnosed with metastatic UM and treated at Veneto Institute of Oncology IOV-IRCCS in Padova, from March 2006 to July 2019. Last follow-up date was September 2020. The biopsies of the 17 liver and 4 extra-hepatic UM metastases included in this study were obtained prior the beginning of treatments. After the diagnosis of UM metastasis, the sequence of treatments varied according to the period of the treatment, the availability of immune checkpoint inhibitors in Italy, the sites of metastases and the clinical conditions of the patients. All patients with a prevalent disease burden in the liver received transcatheter arterial chemoembolization with microbeads charged with irinotecan 100 mg (DEBIRI-TACE). The procedure was repeated 6 weeks later in all patients, and thereafter every 3–6 months according to the disease evaluation. Systemic therapies were administered concomitantly to the liver-directed treatments avoiding the 4 days before and the 10 days after DEBIRI-TACE, to limit the risk of an increased liver toxicity. All participants signed a written informed consent form to allow the use of their diagnostic tumor biopsy for the assessment of tumor immune-microenvironment, and its correlation with the clinical tumor response and outcome. The study was conducted at the Veneto Institute of Oncology IOV-IRCCS in accordance with Good Clinal Practices, local regulatory requirements, and Declaration oh Helsinki.

### Tissue samples

All cases were reviewed and the diagnoses confirmed in all instances by an expert pathologist. Consecutive 4 μm-thick sections were cut from formalin-fixed and paraffin-embedded (FFPE) tissue block of each case for subsequent analyses.

### Multiplex fluorescence immunohistochemistry (mIHC)

mIHC staining was performed using the Opal seven-color manual kit (Akoya Biosciences) following the manufacturer’s instructions, as previously reported [29]. Two staining panels were used to characterize the subsets of tumor-infiltrating immune cells, and the list of antibodies used is summarized in Table 1. All stains were performed under optimized conditions. Before proceeding with multiplex experiments, the optimal staining conditions for each marker of the panel were determined using monoplex stained slides from a positive control tissue (human tonsil). Thus, the antigen-fluorophore pairing (according to the expression characteristics of each specific antigen), the order of primary antibodies addition to the panel (based on the epitope biology and sensitivity), and the concentration and incubation time of primary antibodies and fluorophores were optimized. These parameters were then revaluated in a multiplex stained metastatic UM sample slide, and the optimized multiplex staining protocol was then applied to all sample slides. Moreover, an unstained tissue section was used to subtract the tissue autofluorescence from multiplex-stained slides.

**Table 1:** List of primary antibodies used in mIHC staining

Antigen	Panel	Clone	Vendor
Neutrophil Elastase (NE)	1st	NP57	Dako
CD56	1st	NP57	Dako
CD3	1st	F.7.2.38	Dako
CD20	1st	L26	Dako
CD68	1st	KP1	Dako
CD8	2nd	C8/144B	Dako
CD4	2nd	4B12	ThermoFisher
Granzyme B	2nd	11F1	Leica Biosystems
FoxP3	2nd	D2W8E	Cell Signalling
CD163	2nd	10D6	Leica Biosystems
Anti-melanoma mix (HMB-45 + Mart-1 + Tyrosinase + SOX-10)	1st and 2nd	HMD45 + M2-7C10 + M2-9E3 + T311 + EP268	Abcam and Cell Marque
Spectral DAPI	1st and 2nd		Akoya Biosciences

### Multispectral imaging and analysis

**Fig. 1:**
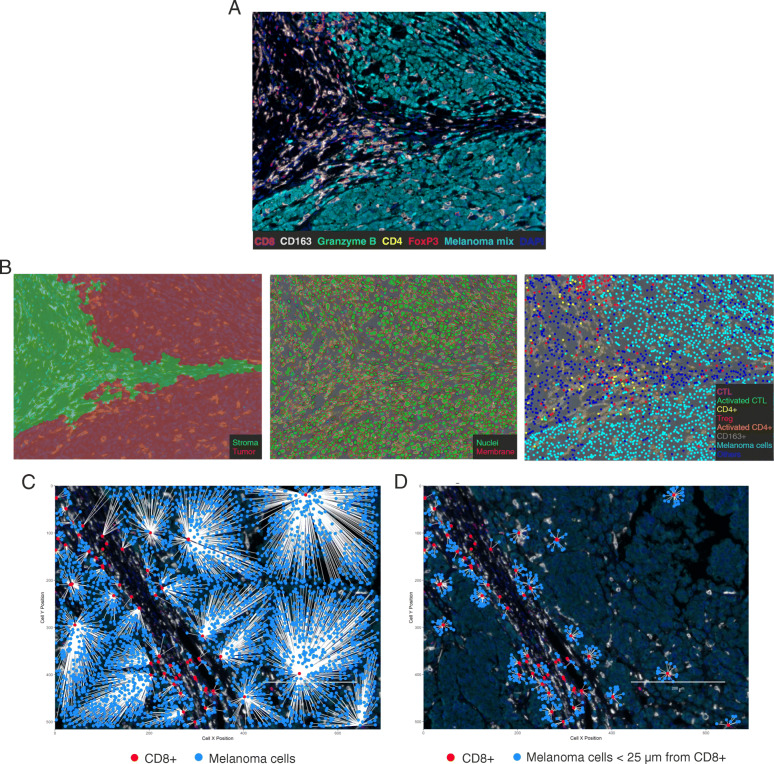
Multispectral imaging and analysis. **a** Representative image of 7-color mIHC staining of a metastatic UM sample scanned with Mantra Quantitative Pathology Workstation at original magnification 20X. Color code is under the picture: CD4 marker is represented in yellow, CD8 in magenta, Granzyme B in green, FoxP3 in red, CD163 in white, melanoma mix in cyan, nuclei in blue. **b** Analysis workflow of acquired multispectral images: (**b**, left) tissue segmentation to differentiate tumor area (in red) and the surrounding stroma (in green); (**b**, middle) single cell segmentation (nuclei are in green, membrane in red); (**b**, right) cell phenotyping based on the detection of specific cell-surface or intracellular markers. **c-d** Representative image of cell-to-cell distance analyses: **c** the nearest neighbor analysis used for the determination of the mean cell distance between melanoma cells (light blue dots) and the nearest CD8+ cells (red dots); **d** count within analysis used for the calculation of the percentage of melanoma cells (light blue dots) within a specified μm radius from a CD8+ T lymphocyte (red dots)

Multiplex stained slides were imaged using the Mantra Quantitative Pathology Workstation (Akoya Biosciences) at 20X magnification (Fig. 1a). For each sample, only areas comprising tumor cells were considered, and at least 20 fields at 20X magnification were acquired for each slide in order to encompass all UM metastasis regions avoiding overlaps between tissue fields. The inForm Image Analysis software (version 2.4.9, Akoya Biosciences) was used to unmix multispectral images using a spectral library built from acquisition of single fluorophore-stained control tissues and containing fluorophores-emitting spectral peaks. A selection of representative multispectral images was used to train the inForm software to create an algorithm for each panel. Tumor tissue was segmented based on recognition of cells stained positive for the anti-melanoma antibody cocktail, to differentiate infiltrating immune cells within the tumor area and in the surrounding stroma (Fig. 1b, left). Then, nuclear counterstaining was used to segmented single cells (Fig. 1b, middle) and cell phenotyping was based on the detection of specific cell-surface or intracellular markers (Fig. 1b, right). The created algorithms were applied in the batch analysis of all acquired seven-color multispectral images of the same panel. Cell densities and percentages were calculated for each patient as the mean of all acquired field of the same tissue slide (at least 20 fields at 20X magnification for each stained slide).

### Cell-to-cell distance analysis

Topographic coordinates for each cell within each tissue section were obtained by InForm software, and distance analyses were performed using Phenoptr and Phenoptr Reports (add-ins for R Studio from Akoya Biosciences). For mean cell distance between tumor cells and the nearest T lymphocyte, the nearest neighbor analysis was used (Fig. 1c), while count within analysis was employed to calculate the percentage of reference cells (tumor or immune cells) within a specified μm radius from a specific immune cell subtype, among the total number of reference cells (Fig. 1d).

### Statistical analysis

All statistical analyses were performed using the GraphPad Prism software (version 7.0). Disease control rate (DCR) was defined as the proportion of patients with stable disease and complete/partial response to treatment, according to RECIST 1.1 criteria [30]. For continuous variables, median, quartiles and range were described and statistical analyses were performed with the non-parametric two-tailed Mann-Whitney test between groups of interest. OS was the time from metastatic UM diagnosis to death. Patients who did not develop an event during the study period were censored at the date of last follow up. For survival analyses, each marker was categorized as low or high according to its median value. The survival probabilities were estimated by the Kaplan-Meier method, and compared between the marker groups using the log-rank Mantel-Cox test. Results were expressed as hazard ratios (HR) with their 95% confidence intervals (95% CI), obtained from a Cox Proportional Hazards model. For the correlation analyses, the non-parametric Spearman’s correlation coefficient (r) was calculated. A *p*-value ≤0.05 was considered statistically significant.

## Results

### Patients characteristics

The characteristics of the 21 patients are summarized in Table 2. Fifteen patients (71.4%) developed metastases confined to the liver, two patients (9.6%) metastasized also in other sites (but only their liver metastases could be evaluated), while in four patients (19%) only extra-hepatic sites were involved. About this latter group, only ureter, lymph node, small intestine and thigh metastases were collected and analyzed. In the study cohort, 13 patients (61.9%) were treated with checkpoint inhibitors immunotherapy, while 8 patients (38.1%) with other targeted/systemic therapies. At a median follow-up of 25.8 months (range 6.8–171) from metastatic UM diagnosis, 11 patients (52.4%) were alive with residual disease, while 10 (47.6%) were dead. Median OS from metastatic UM diagnosis was 95.7 months. Seven patients (33.3%) had progressive disease, 7 patients (33.3%) stable disease, 6 patients (28.6%) reached a partial response and 1 patient (4.8%) obtained a complete response.

**Table 2:** Patient characteristics

	N	%
**Gender**		
Female	9	42.9
Male	12	57.1
**Age at diagnosis of primary UM, years**		
Median (IQR)	61 (49–66)	
**Age at diagnosis of UM metastasis, years**		
Median (IQR)	65 (57–70)	
**Metastasis site**		
Liver	17	81.0
Extra-hepatic	4	19.0
**Liver involvement (*n*** **= 17), %**		
< 20	8	47.1
20–50	8	47.1
> 50	1	5.8
**LDH**		
Normal	15	71.4
> 1ULN	6	28.6
**Treatment**		
Checkpoint inhibitors immunotherapy	13	61.9
Targeted/Systemic therapies	8	38.1
**Response**		
PD	7	33.3
SD	7	33.3
PR	6	28.6
CR	1	4.8
**Status**		
Alive with disease	11	52.4
Dead	10	47.6

*Abbreviations*: *IQR* interquartile range, *N* normal, *ULN* upper limit of normal, *PD* progressive disease, *SD* stable disease, *PR* partial response, *CD* complete response. Response evaluation for each patient was in accordance with RECIST 1.1 [30]

### The immune microenvironment of UM metastases differs between patients with progressive (PD) and controlled (CD) disease

**Fig. 2:**
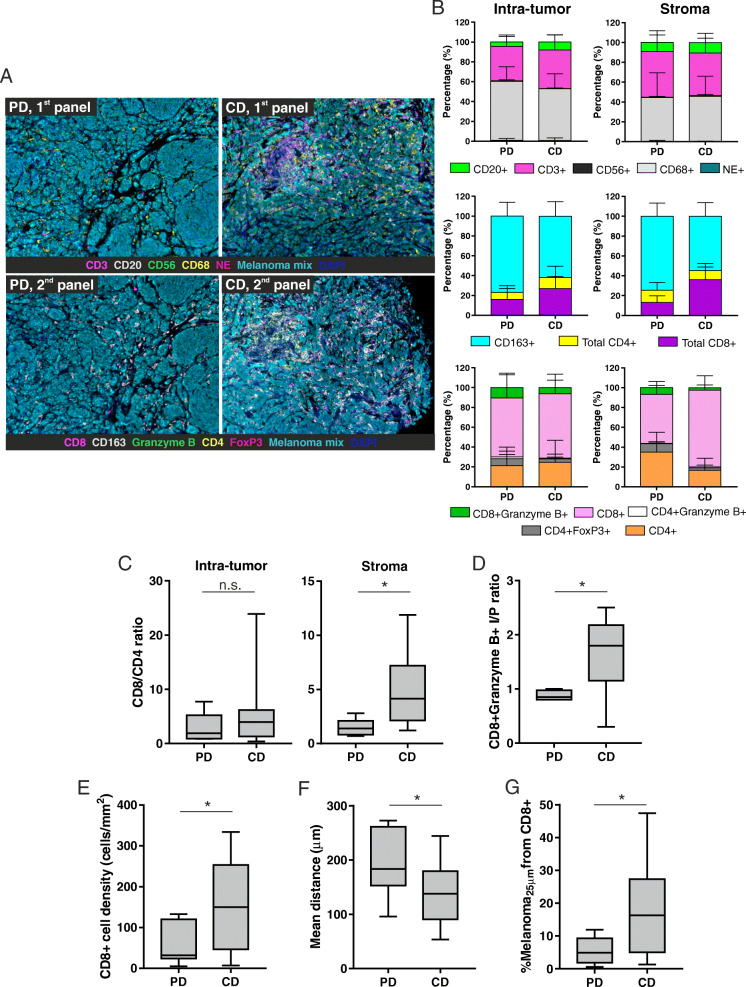
The tumor immune cell composition differs between PD and CD patients. **a** Representative images of 7-color mIHC staining of metastatic UM samples derived from PD and CD patients. First panel (above): CD3 in magenta, CD20 in white, CD68 in yellow, neutrophil elastase in red, CD56 in green, melanoma mix in cyan, nuclei in blue. Second panel (below): CD4 in yellow, CD8 in magenta, Granzyme B in green, FoxP3 in red, CD163 in white, melanoma mix in cyan, nuclei in blue. Original magnification 20X. **b** Immune cell quantitation from the two mIHC panels plotted as the percentage of cells in PD and CD patients, calculated in the intra-tumoral and stromal regions. The mean values and the standard deviations are represented. **c** CD8+/CD4+ cell ratio calculated in the intra-tumoral and stromal compartments in PD and CD patients. **d** Intra-tumoral/Peri-tumoral (I/P) ratio of CD8 + Granzyme+ T lymphocytes in PD and CD patients. **e** Intra-tumoral CD8+ T cells density (number of cells/mm^2^) in PD and CD patients. **f** Mean distance (μm) between each UM cell (stained positive for the melanoma cocktail of antibodies) and the nearest CD3+ T lymphocyte in PD and CD patients. **g** Percentage of UM cells within a radius of 25 μm from CD8+ T lymphocytes in PD and CD patients. Floating box extends from 25th to 75th percentiles, line through the box indicates median, and bars extend from the smallest to largest values. Non-parametric Mann-Whitney statistical analysis was performed across the two groups, and significantly different data are represented by *(*p* < 0.05)

A mIHC approach was applied to FFPE tissue sections of metastatic UM. Two panels of representative immune markers were designed to describe the composition of immune cells infiltrating the metastasis microenvironment. The first panel included CD20, CD3, CD68, CD56 and NE as markers of B lymphocytes, T cells, macrophages, NK cells and neutrophils, respectively. The purpose of the second panel was to investigate the functional state of immune cells, as it included CD4 as a marker of T helper lymphocytes, FoxP3 expressed by CD4+ T regulatory cells (T_reg_, CD4 + FoxP3+), CD8 for cytotoxic T lymphocytes (CTL), granzyme B to identify antitumor activated CTL (CD8+/CD4+ Granzyme B+), and CD163 recapitulating M2-polarized TAM. In both panels, a cocktail of anti-melanoma antigen-recognizing antibodies was added to outline tumor cells (Fig. 2a).

Patients were divided into two subgroups according to the disease control rate: patients with progressive disease (PD, *n* = 7) versus those who had response or stable disease (CD, *n* = 14). In either groups, CD3+ T cells constituted the dominant TIL subset, while CD56+ NK cells and NE+ neutrophils appeared negligible (Fig. 2b, upper panels); CD68+ macrophages were the major cell fraction at intra-tumoral level, being less represented in the peritumoral stroma (Fig. 2b, upper panels).

Notwithstanding, a deeper analysis of macrophage and T cell subpopulations revealed that patients with CD had a lower fraction of CD163+ M2-polarized macrophages and a higher percentage of total CD8+ T lymphocytes than PD patients, both in the intra-tumoral area and in the surrounding stroma (Fig. 2b, middle panels). As the CD8/CD4 ratio is often used as a simple measure to determine the overall balance of T cell function in cancer [31, 32], such lymphocyte subsets were specifically quantified. The total CD4+ T cell component was substantially similar in PD and CD patients irrespectively of the tumor/stroma location; therefore, the CD8/CD4 T cell ratio differed between the two groups, being higher in CD patients mainly in the stromal area (Fig. 2b, middle panels and Fig. 2c). Apart quantitative aspects, sample tissue staining with the second mIHC antibody panel provided further insights about the functional state of T lymphocytes present in the metastatic microenvironment. CD patients had a lower percentage of CD4 + FoxP3+ T_reg_ cells than PD counterparts both in the intra-tumoral and stromal regions, while no difference was observed in the CD4 + Granzyme B+ subset (Fig. 2b, bottom panels). As tumor infiltration by activated CD8+ T lymphocytes is considered a feature associated with tumor-specific immune response, the microenvironment localization of such population was investigated. The CD8 + Granzyme B+ CTL fraction was percentually similar in the intra-tumoral area of either patient groups, but was globally higher in patients with PD whether considering the surrounding stroma (Fig. 2b, bottom panels). On the other hand, a higher CD8 + Granzyme B+ T cell intra-tumoral/peritumoral (I/P) ratio was observed in CD patients (Fig. 2d), suggesting their preferential infiltration of tumor masses where they might be regarded as exerting a direct cytotoxic activity against metastatic UM cells.

Additionally, we calculated the densities of immune cell subsets infiltrating the UM metastases in different areas of tumor microenvironment. No differences were observed in B lymphocytes and myeloid cells (data not shown). However and with regard to the T cell compartment, a higher density of intra-tumoral CD8+ T cells was observed in patients with CD (Fig. 2e).

To assess whether the increased immune infiltrate might associate to inhibitory or activating characteristics, correlation analyses were performed between functionally different immune cell populations. A direct correlation between intra-tumoral CD4+ T lymphocytes and CD4 + FoxP3+ T_reg_ cell densities was observed in PD patients (*r* = 0.9316, 95%CI 0.598 to 0.99; Supplementary Figure 1A), while a positive correlation between intra-tumoral CD4+ T cells and the activated CD4 + Granzyme B+ cell counterpart existed in CD individuals (*r* = 0.6492, 95%CI 0.164 to 0.886; Supplementary Figure 1B), but not in PD patients (*r* = − 0.4714, 95% CI − 0.903 to 0.436; data not shown). Conversely, an inverse correlation between intra-tumoral CD4+ lymphocytes and the percentage of T_reg_ cells was detected in CD patients (*r* = − 0.5867, 95%CI − 0.856 to − 0.064; Supplementary Figure 1C). Moreover, in this latter group, the densities of macrophages (CD68+ and CD163+) and T lymphocytes subpopulations were directly proportional both at intra-tumoral (*r* = 0.6879, 95%CI 0.231 to 0.896 and *r* = 0.7934, 95%CI 0.4399 to 0.934, respectively; Supplementary Figure 1D) and stromal levels (*r* = 0.7566, 95%CI 0.305 to 0.0.93 and *r* = 0.9364 95%CI 0.759 to 0.984, respectively; Supplementary Figure 1E). No association between T cell and macrophage densities was present in PD patients (data not shown). These observations suggest an active immune response that in CD patients is mediated by both T and myeloid cells.

To explore the spatial distributions and interactions of cancer cells with respect to immune cells, the Cartesian coordinates were calculated for each cell subset within each tissue section, and cell-to-cell distance analyses were performed (Fig. 1c, d). As the close proximity between T lymphocytes and tumor cells is held as an indicator of an ongoing specific anti-tumor immune response, the nearest neighbor analysis for phenotype pairs was carried out to calculate the mean distance from each melanoma cell (stained positive for the melanoma cocktail of antibodies) to the nearest CD3+ T lymphocyte. Patients with CD showed a shorter average distance between tumor and T cells than patients having PD (Fig. 2f). Considering the lymphocyte and melanoma cell dimensions, a 25–30 μm distance between cells is indicative of an enhanced probability for cell-to-cell contact [33]. CD patients had a significantly increased percentage of UM cells within a radius of 25 μm from CD8+ T cells, as compared to PD patients (Fig. 2g). Although not significant, CD patients also showed a trend for a higher frequency of melanoma cells within a 30 μm radius from CD8 + Granzyme B+ activated CTL than PD patients (Supplementary Figure 2).

### The tumor immune cell contexture differs between liver (LM) and extra-hepatic (EM) UM metastases

To assess whether differences existed in the microenvironment cell composition of hepatic and extra-hepatic UM metastases, patients were divided into two groups according to the site of liver (LM *n* = 17) or extra-hepatic (EM *n* = 4; Table 1) metastasis, to be subsequently analyzed by mIHC. Two patients had both hepatic and extra-hepatic metastases, but they were included in the LM group as only their hepatic metastases were evaluated.

**Fig. 3:**
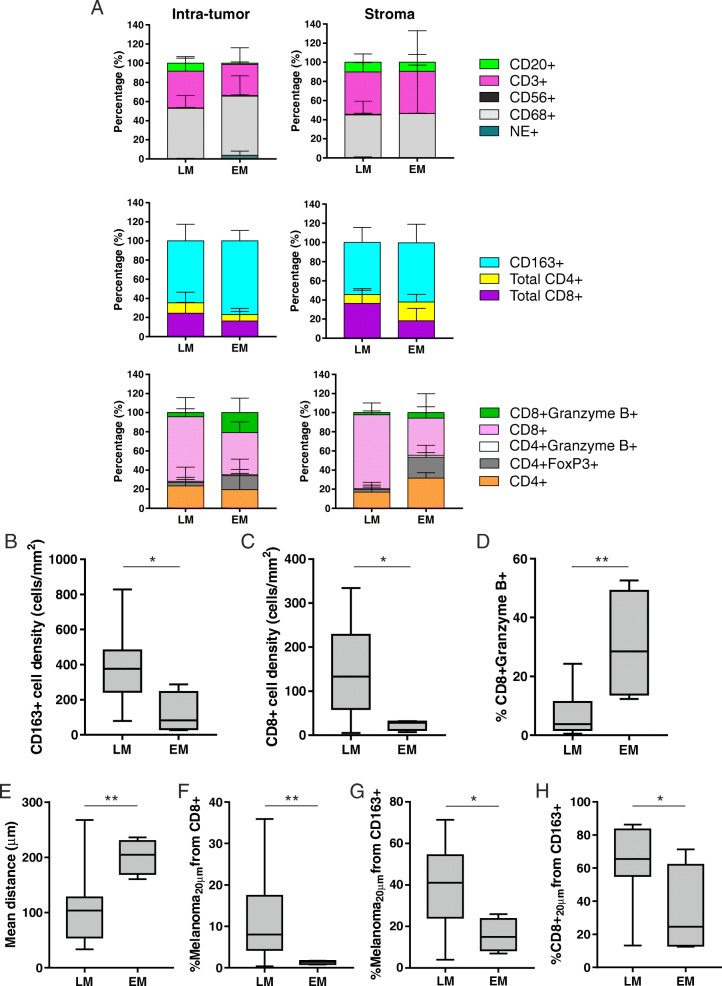
The tumor immune cell composition differs according to the site of UM metastases. **a** Immune cell quantitation from the two mIHC panels plotted as percentage of cells in LM and EM, calculated in the intra-tumoral and stromal regions. The mean values and the standard deviations are represented. **b-c** Intra-tumoral cell densities (number of cells/mm^2^) of CD163+ M2-polarized macrophages (**b**) and CD8+ T lymphocytes (**c**) in LM and EM. **d** Percentage of intra-tumoral activated CD8 + Granzyme B+ T lymphocytes among intra-tumoral total CD8+ cells in LM and EM. **e** Mean distance (μm) between each UM cell (stained positive for the melanoma cocktail of antibodies) and the nearest CD8+ T lymphocyte in LM and EM. **f-g** Percentage of UM cells within a radius of 20 μm from CD8+ T lymphocytes (**f**) and from CD163+ TAMs (**g**) in LM and EM. **h** Percentage of CD8+ T lymphocytes within a radius of 20 μm from CD163+ TAMs in LM and EM. Floating box extends from 25th to 75th percentiles, line through the box indicates median, and bars extend from the smallest to largest values. Non-parametric Mann-Whitney statistical analysis was performed across the two groups, and significantly different data are represented by *(*p* < 0.05), **(*p* < 0.01)

Although the CD3+ T cell lineage was the most represented in the TIL population in either groups and irrespective of location, LM patients disclosed a higher percentage of intra-tumor B and T lymphocytes as compared to patients with EM (Fig. 3a, upper panels). These latter, on the other hand, showed a higher percentage of intra-tumoral macrophages (both CD68+ and CD163+; Fig. 3a, upper and middle panels). Whether considering the T cell composition of the intra-tumoral compartment, in either group of patients total CD8+ cells were more represented than CD4+ cells (Fig. 3a middle panels), but their activation status was different. Indeed, LM patients showed a lower percentage of CD8 + Granzyme B+ lymphocytes and T_reg_ cells (Fig. 3a bottom panels). In the peritumoral stroma, patients with LM displayed a 2-fold increased percentage of CD8+ T cells than EM patients, but an about 50% reduction in CD4+ T helper cells (Fig. 3a middle panels). As in the intra-tumoral region, also in the stroma activated CTL and T_reg_ cells were less represented in LM as compared to EM (Fig. 3a bottom panels). CD4 + Granzyme B+ T cells and NK cells were negligible (Fig. 3a).

Quantitative analysis showed that the intra-tumoral densities of CD163+ M2-polarized macrophages and total CD8+ T cells were higher in patients with LM as compared to those with EM (Fig. 3b, c). Conversely, the percentage of CD8 + Granzyme B+ activated CTL among the total CD8+ population within the tumor compartment was lower in LM patients (Fig. 3d).

A direct correlation between T and B cells was observed in both the intra-tumoral (*r* = 0.615, 95% CI 0.176 to 0.85) and peri-tumoral regions of LM (*r* = 0.548, 95% CI 0.056 to 0.826; Supplementary Figure 3A). Moreover, also T cell and macrophage densities were directly proportional in these areas (*r* = 0.641, 95% CI 0.217 to 0.861 within the tumor and *r* = 0.711, 95% CI 0.319 to 0.895 in the stroma; Supplementary Figure 3B). Of note, when considering only the M2-polarized fraction of macrophages, the association with CTL was observed only within the tumor masses (*r* = 0.537, 95% CI 0.06 to 0.814; Supplementary Figure 3C). Furthermore, an inverse correlation between the percentage of activated CTL and the density of CD8+ cells (*r* = − 0.531, 95% CI − 0.79 to 0.006) or CD163+ cells (*r* = − 0.653, 95% CI − 0.867 to − 0.237) was disclosed in the intra-tumoral area (Supplementary Figure 3D). Collectively, these findings suggest that CTL may drive the recruitment of immunosuppressive macrophages to the liver [34], and that this TAM subset may contribute to limit a potentially antitumor immune response in UM-derived LM.

To validate these assumptions, spatial metric analyses were performed and cell-to-cell interactions were assessed. In the nearest neighbor distance analysis, a significant lower mean distance from each melanoma cell and the nearest CD8+ T lymphocyte was observed in patients with LM as compared to patients with metastasis in other sites (Fig. 3e). Based on the quantitative analysis performed on the entire tumor microenvironment area, LM patients displayed a higher percentage of melanoma cells within a 20 μm radius from CD8+ CTL (Fig. 3f). However, this group of patients showed also an increased frequency of tumor cells within a 20 μm radius from CD163+ M2-polarized macrophages (Fig. 3g), and a higher percentage of CD8+ T lymphocytes within a 20 μm radius from CD163+ TAM (Fig. 3h). Collectively, these features are highly suggestive of a role for TAM in compromising the antitumor immune response in UM liver metastases.

### The immune cells density impacts on overall survival of patients with UM metastases

Distance analysis carried out in patients based on their status at the last follow-up time point, revealed that alive patients (*n* = 11) had a higher frequency of melanoma cells within a 30 μm radius from CD8 + Granzyme B+ CTL, in comparison to dead patients (*n* = 10; Table 2 and Supplementary Figure 4). This prompted further exploration on the impact of immune cell subtypes populating the tumor microenvironment on survival.

**Fig. 4:**
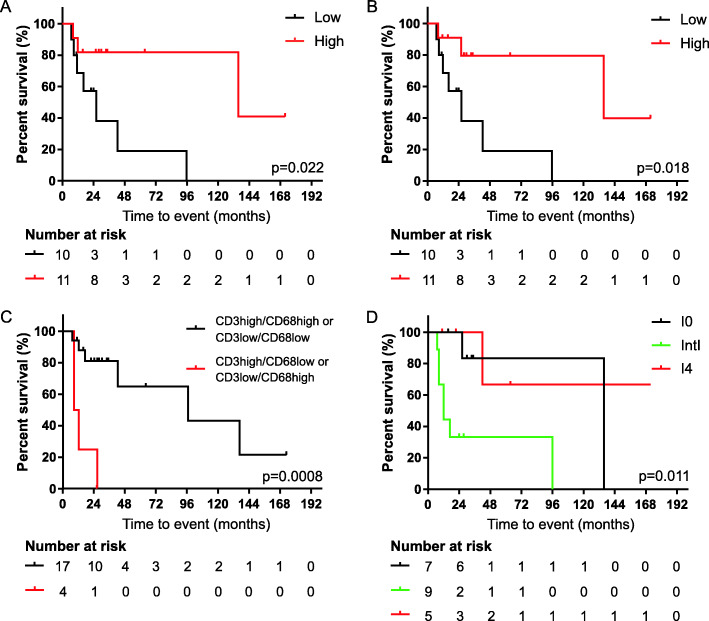
Effect of immune cell infiltrates on overall survival of metastatic UM patients. **a-b** Kaplan-Meier curves for overall survival according to the percentage of activated CD8 + Granzyme B+ T lymphocytes among the total CD8+ cells, calculated in the total area (**a**) or only in the intra-tumoral region (**b**). The median cutoff of CD8 + Granzyme B+ percentage was used to separate high and low infiltrate groups. **c** Kaplan-Meier curves for overall survival according to the combination of intra-tumoral CD3+ T lymphocytes and CD68+ macrophages densities. The median cutoff of each immune cell subset density was used to separate high and low infiltrate. **d** Evaluation of the Immunoscore as a prognostic biomarker in metastatic UM. Kaplan-Meier curves for overall survival according to Immunoscore: Immunoscore 0 (I0, *n* = 7); Immunoscore 1,2,3 (IntI, *n* = 9); Immunoscore 4 (I4, *n* = 5). Log-rank statistics were performed to determine significance, *p* values and the number of patients at risk for each time point are reported in each graph

Based on the median cell densities and cell percentages of each immune cell subset, UM metastases were stratified into high or low tumor-infiltrated groups. The analysis was then performed considering the total area examined (tumor core and peritumoral stroma), the intra-tumoral region only, and the peritumoral stroma only. The density of any infiltrating T cell or macrophage subtype did not significantly associate with survival (data not shown). Notwithstanding and whether considering the total area of tumor microenvironment, a high percentage of activated CD8 + Granzyme B+ T lymphocytes among total CD8+ T cells was associated with a significantly prolonged survival (median OS: 135.6 months in the High group vs. 26.2 months in the Low group; HR 0.24, 95% CI 0.06–0.89, *p* = 0.022; Fig. 4a). As we previously reported that also the immune effector cell localization in different areas of tumor microenvironment is an important determinant of patient outcome (Fig. 2), we separately assessed the prognostic value of stromal and intra-tumoral activated CTL. A significant prognostic value was obtained considering only the percentage of intra-tumoral CD8 + Granzyme B+ CTL, as patients with high percentages of such activated CD8+ cells had longer OS (HR 0.23, 95% CI 0.06–0.86, *p* = 0.018; Fig. 4b). These data support the importance of activated CTL tumor infiltration in the prognosis of patients with metastatic UM. Tertiary lymphoid structures (TLS) were also searched and found in 6 out the 21 (28.5%) metastatic UM samples, but their presence did not associate with OS (Supplementary Figure 5).

Moreover, as the evaluation of the single biomarkers density did not correlate with patient outcome, we explored a combined assessment of both the density and the spatial distribution of the two immune cell populations mainly involved in tumor dynamics, namely T lymphocytes (CD3+ cells) and macrophages (CD68+ cells). Given the small number of patients analyzed and the observation that the activation of lymphoid and myeloid response correlated each other in patients with CD (Supplementary Figure 1), two groups were created: one consisted of UM metastases with both CD3+ and CD68+ cell densities respectively higher (CD3high/CD68high) or lower (CD3low/CD68low) than the corresponding median values. The second group comprised the UM metastases with discordance between lymphoid and myeloid cell densities (CD3high/CD68low and CD3low/CD68high). Considering the peritumoral stroma, no significant difference was observed between the two groups (data not shown). Within the tumor region, however, patients with the concordance between T cell and macrophage densities (CD3high/CD68high and CD3low/CD68low) exhibited a significant prolonged OS compared to patients with CD3high/CD68low and CD3low/CD68high (median OS: 96 months vs 10.2 months, respectively; HR 0.12, 95% CI 0.003–4.73, *p* = 0.0008; Fig. 4c). These results highlight the requirement for a balanced lymphoid and myeloid response in the tumor microenvironment of metastatic UM for a better outcome.

Moreover, in an attempt to provide a more comprehensive picture about the importance of the amount of the T cell infiltrate in relation to survival, a temptative Immunoscore was generated by considering the median densities of CD3+ and CD8+ T cell subtypes in both the intra-tumoral region and the peri-tumoral stroma. Patients with infiltrating T lymphocyte densities lower than the corresponding median value were scored as Immunoscore 0 (I0), while those with both CD3+ and CD8+ cell densities within tumor and stroma regions higher than the median values were scored as Immunoscore 4 (I4). The remaining patients with an Immunoscore ranging from 1 to 3 were grouped and scored as Intermediate Immunoscore (IntI). Kaplan-Meier curves showed three distinct patient groups with statistical differences in OS (*p* = 0.011; Fig. 4d). Notably, patients with IntI experienced the worst outcome, with a median survival of 12 months when compared to patients with I0 (median survival: 135.6 months, HR 5.0, 95% CI 1.32–18.8, *p* = 0.015) and those with I4 (HR 5.9, 95% CI 1.48–23.9, *p* = 0.044; Fig. 4d).

### The tumor immune contexture is associated with overall survival in immunotherapy-treated patients

**Fig. 5:**
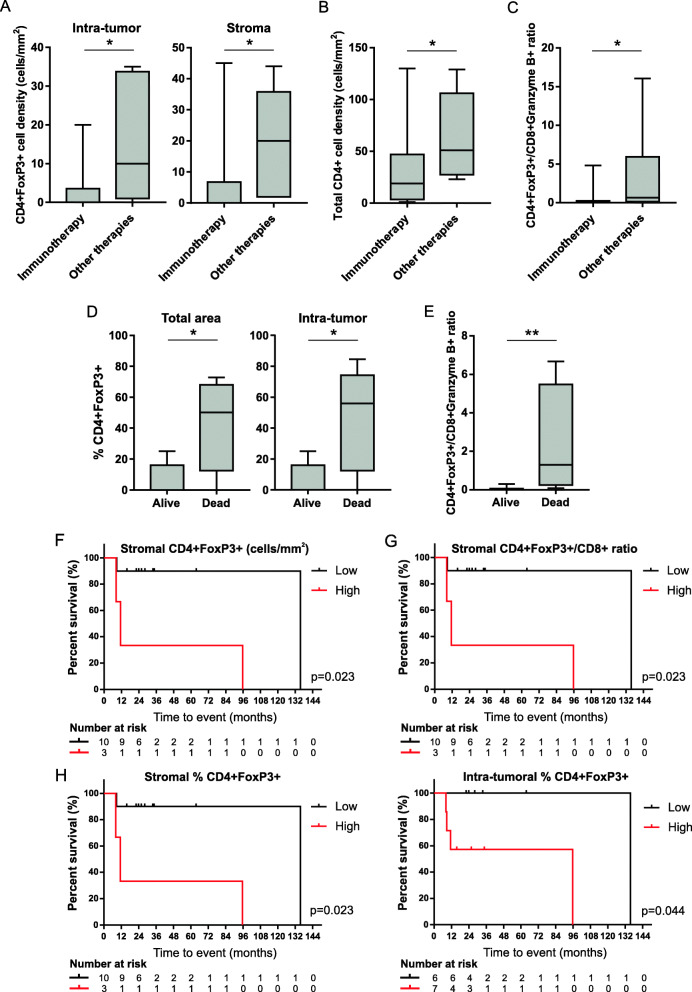
The impact of the tumor immune contexture on overall survival of immunotherapy-treated patients. **a-c** Comparison between immunotherapy-treated patients and individuals receiving other therapies in terms of (**a**) intra-tumoral and stromal CD4 + FoxP3+ T_reg_ cell densities (number of cells/mm^2^), (**b**) intra-tumoral total CD4+ T cell density (number of cells/mm^2^), and (**c**) intra-tumoral CD4 + FoxP3+/CD8 + Granzyme B+ cell ratio. **d** Percentage of CD4 + FoxP3+ cells among total CD4+ lymphocytes, and (**e**) CD4 + FoxP3+/CD8 + Granzyme B+ cell ratio in the total area of immunotherapy-treated patients who were alive or death at the end of the follow-up. Floating box extends from 25th to 75th percentiles, line through the box indicates median, and bars extend from the smallest to largest values. Non-parametric Mann-Whitney statistical analysis was performed across the two groups, and significantly different data are represented by *(*p* < 0.05) and **(*p* < 0.01). **f-h** Kaplan-Meier overall survival curves in immunotherapy-treated patients according to (**f**) the stromal CD4 + FoxP3+ cell density, **g** the stromal CD4 + FoxP3+/CD8+ cell ratio, and (**h**) the percentage of CD4 + FoxP3+ cells among total CD4+ lymphocytes calculated in the stromal or intra-tumoral regions. Log-rank statistics were performed to determine significance, *p* values and the number of patients at risk for each time point are reported in each graph

In the study cohort, 13 patients (61.9%) were treated with checkpoint inhibitors immunotherapy, while 8 patients (38.1%) with other targeted/systematic therapies (Table 2). No difference in the OS was observed between the two groups (*p* = 0.43), although immunotherapy-treated patients displayed a significantly lower density of CD4 + FoxP3+ T_reg_ lymphocytes both in the intra-tumoral and stromal regions, as compared to individuals receiving other treatments (Fig. 5a). Moreover, within the tumor region, they had a lower density of total CD4+ cells (Fig. 5b) and a lower CD4 + FoxP3+/CD8 + Granzyme B+ cell ratio (Fig. 5c). Whether considering only the proportion of patients treated with immunotherapies, we additionally observed that those still alive at the end of the follow-up had a lower percentage of T_reg_ cells both in the total area and in the intra-tumoral region only (Fig. 5d), and a lower CD4 + FoxP3+/CD8 + Granzyme B+ cell ratio (Fig. 5e). Finally, in immunotherapy-treated patients the low density of stromal T_reg_ cells (Fig. 5f), the low stromal T_reg_/CD8+ cell ratio (Fig. 5g), and the low percentage of T_reg_ cells among total CD4+ T lymphocytes both at stromal and intra-tumoral level (Fig. 5h), are all features associated with a prolonged OS. Collectively, these observations suggest that CD4 + FoxP3+ T_reg_ cells appear a crucial population for response to immunotherapy.

## Discussion

Currently, the present study comprises one of the largest sample cohort in which the metastatic UM immune microenvironment has been quantitatively analyzed, and in which the frequency and composition of immune cell infiltrate is correlated with patient outcome. Moreover, also the cell topography and thereby the probability of cell-to-cell interactions has been investigated, with additional correlations to clinical and prognostic parameters.

The eye is considered an immune-privileged site, and the immunobiology of primary UM has been already object of extensive investigation [21, 23, 35, 36]. However, only few studies have focused on the characterization of the immune infiltrate and microenvironment of metastatic UM [24, 25, 37], mainly due to the limited availability of biological samples, and because the vast majority of patients are not qualified for surgical resection due to the number or distribution of lesions [38]. This aspect is critical for single-marker IHC studies, which often lead to incomplete quantitative data due to the exhaustion of the FFPE blocks [39]. Multispectral imaging performed on metastatic UM samples allowed us to objectively assess seven markers simultaneously, to precisely quantify the number of cells with a specific phenotype and to determine their cartographic coordinates on a single 4-μm FFPE tissue section, thus consuming very few amounts of the precious metastatic UM sample.

Our observations on immune cells infiltrating UM metastases are consistent with recently published studies [24, 25, 37], but we have additionally found that the immune cell subsets composition differs according to patient response. Indeed, in UM metastases from PD patients we identified immune features suggestive of an impaired antitumor immune response, such as a relevant presence of pro-tumorigenic M2 macrophages and T_reg_ cells, a reduced intra-tumoral CTL density and a lower stromal CD8/CD4 ratio. These results are in line with the observation that *NRP1* gene, which is involved in the immune-modulation of T_reg_ cells and M2-polarized macrophages, is upregulated in metastatic UM patients with an OS less than 1 year [40]. Moreover, the spatial context of immune cells has been shown to be critical for cancer development [41], since effector cells require close contacts with target cells to exert their cytotoxic antitumor functions. Our observations that the percentage of melanoma cells close to T cells was higher in CD than PD patients and that the majority of CD8 + Granzyme B+ T cells could be detected within the intra-tumoral region, suggest a specific anti-tumor effector role of CTL that may perform important biological functions in metastatic UM.

The potential importance of this cell subset is also supported by the observation that the percentage of activated CTL acts as a prognostic indicator able to stratify metastatic UM patients with better OS, while the mere density of total CD8+ T lymphocytes did not associate with patient outcome. Of note, when discriminating between the intra-tumoral and peri-tumoral regions, only the percentage of CD8 + Granzyme B+ T lymphocytes within the tumor masses retained the prognostic value, supporting the importance of effector cell localization in metastatic UM. All together, these observations go beyond the bias of previous studies that focused only on the overall cell counts using single marker IHC [24, 25], highlighting the clinical relevance and possible functional importance of T cell infiltration for metastatic UM control.

TLS may support the activation of CTL against tumor cells, as the presence of TLS in melanoma patients was associated with improved outcome [42]. However, in tumors arising in immunologically privileged sites, such us the brain (glioblastoma) and the eye (uveal melanoma), TLS are usually infrequent [43]. In agreement with this observation, we found TLS only in a small proportion of metastatic UM patients, and no prognostic value was observed.

The complexity of mechanisms orchestrating the immune response against metastatic UM is underlined by the observation that a delicate equilibrium exists in patients with better outcome between lymphoid and myeloid cell responses within the tumor region, but not in the peri-tumoral stroma. Our data are in line with what observed by Massi et al. in a cohort of 158 metastatic cutaneous melanoma patients treated with MAPK inhibitors [44]. Paradoxically, high densities of TAMs and TILs in primary UM are associated with a poor prognosis [21, 45], likely because of the immunoregulatory influence of the intraocular microenvironment and the macrophage-mediated regulation of angiogenesis and cancer cell migration, which could promote tumor growth. Thus, the association of low densities of both T lymphocytes and macrophages with a prolonged OS in UM metastases, might be reminiscent of the primary tumor microenvironment. These results suggest that the combined evaluation of the density and spatial distribution of CD3+ and CD68+ cells in metastatic UM patients can be used as a prognostic indicator in metastatic UM.

The Immunoscore has been reported to overcome the classical TNM system in predicting disease-free survival (DFS) and OS in colorectal cancer (CRC) [46]. The definition of an Immunoscore in cutaneous melanoma is still challenging, even though it is currently under evaluation in lymph node metastases from stage III melanoma patients [47] and in metastatic tissues from individuals treated with Ipilimumab (the MISIPI study) [48]. In this scenario, we tried to transfer the Immunoscore concept to metastatic UM as a potential prognostic marker. Despite the limited dimension of our cohort, the Immunoscore stratified patients in three distinct groups with statistically significant differences in terms of OS. However, while a low Immunoscore associates with the shortest DFS and OS in CRC patients, were the metastatic UM patients with I0 and I4 to exhibit a significantly increased OS as compared with those having an intermediated Immunoscore. Reasons for this unexpected trend require further investigations in a larger cohort.

Patients with hepatic UM metastases usually experience a worse outcome as compared to individuals with extra-hepatic UM metastases only [10], thus suggesting a role for the microenvironment in UM metastatic progression. In this study, we had the chance to collect not only hepatic but also extra-hepatic UM metastases, and this allowed a comparative analysis of the tumor immune infiltrate between different UM metastatic sites. The liver is considered an immuno-modulatory organ, whose microenvironment could promote UM metastatic growth by protecting melanoma cells from immune surveillance [49]. Differently from what described by Qin et al. [39], we observed differences in the tumor immune cell contexture between hepatic and extra-hepatic UM metastases, supporting the delicate balance between immune elements with anti- or pro-tumorigenic functions in liver UM metastases. Indeed, the high density of intra-tumoral CD8+ T lymphocytes in LM was counterbalanced by an analogous elevated density of CD163+ M2-polarized macrophages, and by a low percentage of CD8 + Granzyme B+ activated CTL. Moreover, although we detected a higher percentage of tumor cells in close proximity to T lymphocytes as compared to EM, more than 60% of CTL in LM were in contact with M2-polarized macrophages, thus suggesting a role of TAM in suppressing cytotoxic CD8+ T cell functions. Furthermore, we observed that a high percentage of UM cells in liver were adjacent to pro-tumorigenic CD163+ TAMs. In melanoma cells, the expression of particular molecules, such as colony-stimulator factor 1 (CSF1) or CD47, in response to T-cell derived cytokines represents a conserved and adaptive resistance mechanism involved in disease progression [34]. Indeed, the interaction between CSF1 on melanoma cells and its CSF1 receptor on macrophages shapes the tumor myeloid cell compartment toward immunosuppression by inducing the differentiation and accumulation of M2 TAM. Besides, the binding of CD47 on cancer cells with the inhibitory receptor signal regulatory protein alpha (SIRPα) on TAM suppresses the ability of macrophages to detect and phagocytose tumor cells [50]. Collectively, these features are highly suggestive of a key role of TAM to impair the antitumor immune response in UM liver metastases by inhibiting the activation of CTL that are recruited to the tumor site. These observations could explain the worse outcome of patients with UM-derived LM and could have implications in the treatment modalities of these patients, through the depletion or re-education of TAM [51].

Finally, we also explored the immune populations indicative of a better response to immunotherapy. Despite the low response rate and the negligible impact on survival of checkpoint inhibitors in metastatic UM [52], in our cohort the patients with a better outcome disclosed a lower percentage of T_reg_ lymphocytes and a lower T_reg_/CTL ratio, thus indicating that the amount of CD4 + FoxP3+ T_reg_ cells in metastatic UM might be considered a predictive biomarker for the response to immunotherapy. In support of this hypothesis, the gene expression analysis performed by Qin et al. on pre-treatment samples from six immunotherapy-treated metastatic UM patients revealed an upregulation of genes encoding cytokines and molecules of the pro-inflammatory signal network regulated by IL-13, IL-4 and NF-κB in non-responding individuals, and the upregulation of IFN-γ-regulated genes (*SOCS1* and MHC) in responding patients [39].

We are aware that this study has some limitations. First, although being one of the biggest collections examined to date, the cohort is still limited. This aspect, together with the most recent case history, could have influenced the longer median OS observed in our cohort, as compared to what found in literature [8, 9]. Second, patients were differently treated after metastatic UM diagnosis due to the different available treatments during the accrual; therefore, a possible influence of treatments on survival could not be ruled out. However, the absence of standard effective therapies should exclude or limit the treatment effect. Third, the expression of immune checkpoint molecules was not assessed, mainly due to the very limited availability of material samples. In this regard, however, there is increasing evidence that UM metastases are characterized by reduced levels of PD-1+ lymphocytes and PD-L1-expressing cells as compared to cutaneous melanoma metastases [37, 39, 53], and this can provide a potential explanation for the failure of immunotherapy in UM [27, 52]. On the other hand, molecular profiling at single cell-resolution on a limited number of UM liver metastases, showed the expression of TIM-3, LAG-3, and to some extent, TIGIT receptors on TILs, thus suggesting that alternative immune checkpoints may play a role in T cell response inhibition [36, 54].

## Conclusions

In conclusion, we demonstrated that a mIHC approach provides a meaningful opportunity to study the interactions and spatial relationships between tumor and different immune cell types, and to describe the complex landscape of metastatic microenvironment in UM, thus helping to identify more effective and personalized treatment strategies. In this study, we correlated the immune composition of UM metastases microenvironment with the disease control rate, the site of metastasis and patient outcome (Supplementary Figure 6). In particular, we observed that i) the immune cell subsets composition differs according to patient response, highlighting the clinical relevance and possible functional importance of T cell infiltration for metastatic UM control (Supplementary Figure 6B). ii) A delicate balance exists between immune elements with anti- or pro-tumorigenic functions in liver UM metastases, which could promote UM metastatic growth by protecting melanoma cells from immune surveillance, ultimately explaining the worse outcome of patients with liver metastasis from UM (Supplementary Figure 6C). iii) The percentage and the tumor localization of activated cytotoxic T lymphocytes act as a prognostic indicator able to stratify metastatic UM patients with better OS (Supplementary Figure 6D). iv) Finally, CD4 + FoxP3+ T cells appear a crucial population for response to immunotherapy (Supplementary Figure 6E).

## Supplementary Information

**Additional file 1: Supplementary Figure 1.** Correlation analyses. Correlation between different immune cell infiltrates in PD **(A)** and CD **(B-E)** patients, within the tumor and in the stroma regions. Data are represented in a scatter plot with the best fit shown as solid line. The non-parametric Spearman’s correlation coefficient (r) and *p* value were calculated for each graph. **Supplementary Figure 2**. Percentage of UM cells within a radius of 30 μm from CD8 + Granzyme B+ T lymphocytes in PD and CD patients. Floating box extends from 25th to 75th percentiles, line through the box indicates median, and bars extend from the smallest to largest values. Non-parametric Mann-Whitney statistical analysis was performed across the two groups. **Supplementary Figure 3**. Correlation analyses. Correlation between different immune cell infiltrates in LM within the tumor and in the stroma regions. Data are represented in a scatter plot with the best fit shown as solid line. The non-parametric Spearman’s correlation coefficient (r) and *p* value were calculated for each graph. **Supplementary Figure 4**. Percentage of UM cells within a radius of 30 μm from CD8 + Granzyme B+ T lymphocytes in patients alive and dead at the last follow-up time point. Floating box extends from 25th to 75th percentiles, line through the box indicates median, and bars extend from the smallest to largest values. Non-parametric Mann-Whitney statistical analysis was performed across the two groups, and significantly different data is represented by *(*p* < 0.05). **Supplementary Figure 5**. The presence of TLS did not correlate with metastatic UM patient’s prognosis. **A)** Representative 7-color mIHC image of a TLS found in a metastatic UM sample. Markers and color code are indicated under the picture. Original magnification 20X. **B)** Kaplan-Meier curves for overall survival according to the presence or absence of TLS in the tumor microenvironment. Log-rank statistics were performed to determine significance, *p* value and the number of patients at risk for each time point are reported. **Supplementary Figure 6**. A schematic cartoon illustrating the key findings of the manuscript.

## Data Availability

Not applicable.

## Abbreviations

UM: Uveal melanoma
OS: Overall survival
CTLA-4: Cytotoxic T-lymphocyte antigen 4
PD-1: Programmed cell death protein 1
TILs: Tumor-infiltrating lymphocytes
TAMs: Tumor-associated macrophages
FFPE: Formalin-fixed paraffin-embedded
mIHC: Multiplex immunohistochemistry
DCR: Disease control rate
HR: Hazard ratio
CI: Confidence interval
LDH: Lactate dehydrogenase
DEBIRI-TACE: Transcatheter arterial chemoembolization with microbeads charged with irinotecan
PD: Progressive disease
SD: Stable disease
PR: Partial response
CR: Complete response
CD: Controlled disease
NK: Natural killer
T_reg_: T regulatory
CTL: Cytotoxic T lymphocytes
LM: Liver metastasis
EM: Extra-hepatic metastasis
I0: Immunoscore 0
I4: Immunoscore 4
IntI: Intermediate Immunoscore
MAPK: Mitogen-activated protein kinase
TNM: Tumor, node, metastasis
CRC: Colorectal cancer
DSF: Disease-free survival
CSF1: Colony-stimulator factor 1
SIRPα: Signal regulatory protein alpha
NRP1: Neuropilin 1
SOCS1: Suppressor of cytokine signaling 1
MHC: Major histocompatibility complex
