# Supplementary material for: The immune cell landscape of metastatic uveal melanoma correlates with overall survival
Source: J Exp Clin Cancer Res. 2021 May 4;40:154. doi: 10.1186/s13046-021-01947-1 (PMC8097926; doi:10.1186/s13046-021-01947-1)
Supplement: Supplementary file 1 — Additional file 1: Supplementary Figure 1. Correlation analyses. Correlation between different immune cell infiltrates in PD (A) and CD (B-E) patients, within the tumor and in the stroma regions. Data are represented in a scatter plot with the best fit shown as solid line. The non-parametric Spearman’s correlation coefficient (r) and p value were calculated for each graph. Supplementary Figure 2. Percentage of UM cells within a radius of 30 μm from CD8 + Granzyme B+ T lymphocytes in PD and CD patients. Floating box extends from 25th to 75th percentiles, line through the box indicates median, and bars extend from the smallest to largest values. Non-parametric Mann-Whitney statistical analysis was performed across the two groups. Supplementary Figure 3. Correlation analyses. Correlation between different immune cell infiltrates in LM within the tumor and in the stroma regions. Data are represented in a scatter plot with the best fit shown as solid line. The non-parametric Spearman’s correlation coefficient (r) and p value were calculated for each graph. Supplementary Figure 4. Percentage of UM cells within a radius of 30 μm from CD8 + Granzyme B+ T lymphocytes in patients alive and dead at the last follow-up time point. Floating box extends from 25th to 75th percentiles, line through the box indicates median, and bars extend from the smallest to largest values. Non-parametric Mann-Whitney statistical analysis was performed across the two groups, and significantly different data is represented by *(p < 0.05). Supplementary Figure 5. The presence of TLS did not correlate with metastatic UM patient’s prognosis. A) Representative 7-color mIHC image of a TLS found in a metastatic UM sample. Markers and color code are indicated under the picture. Original magnification 20X. B) Kaplan-Meier curves for overall survival according to the presence or absence of TLS in the tumor microenvironment. Log-rank statistics were performed to determine significance, p value and the number [file 13046_2021_1947_MOESM1_ESM.pdf]

Supplementary Figures

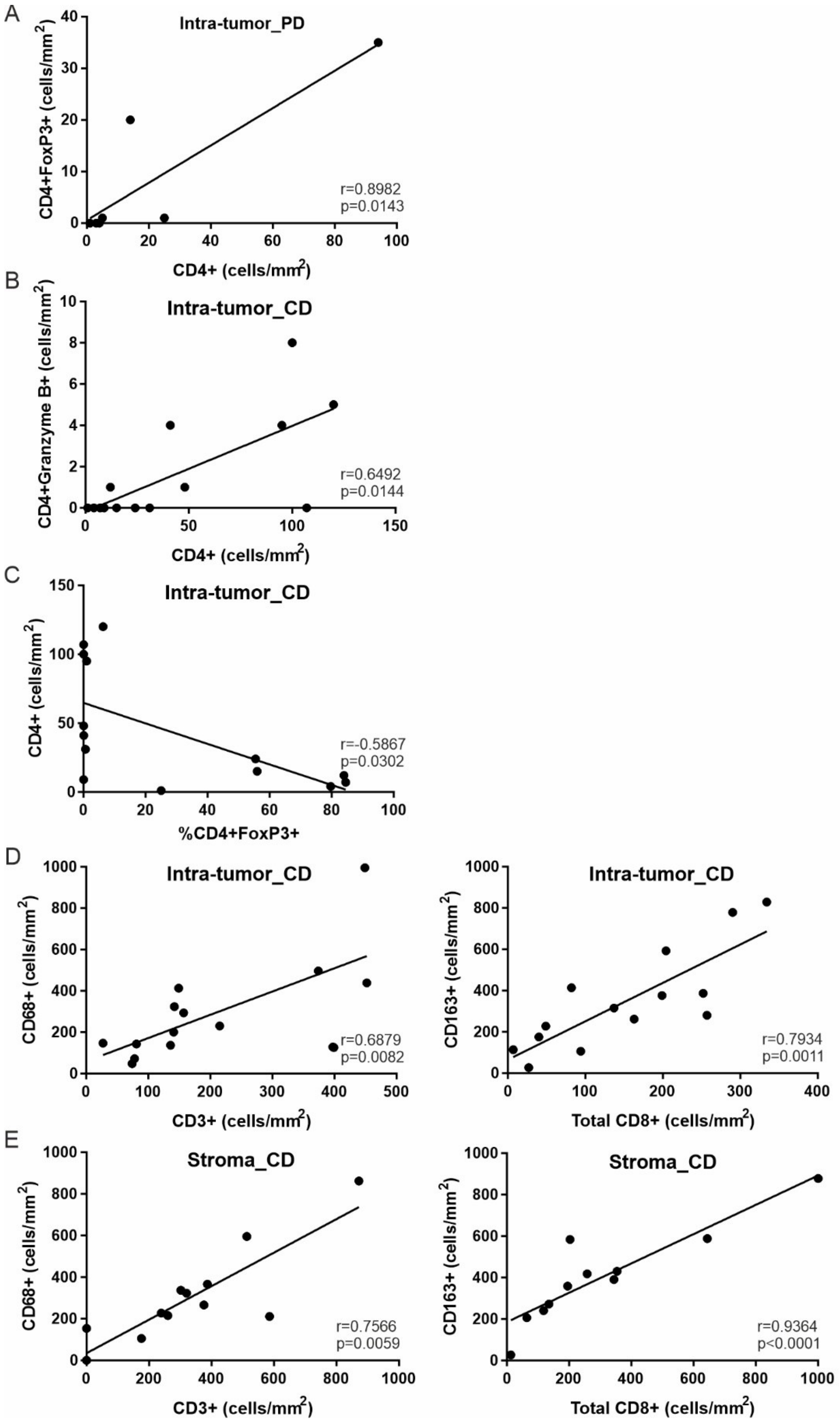

Supplementary Figure 1. Correlation analyses. Correlation between different immune cell infiltrates in PD **(A)** and CD **(B-E)** patients, within the tumor and in the stroma regions. Data are represented in a scatter plot with the best fit shown as solid line. The non-parametric Spearman's correlation coefficient (r) and p value were calculated for each graph.

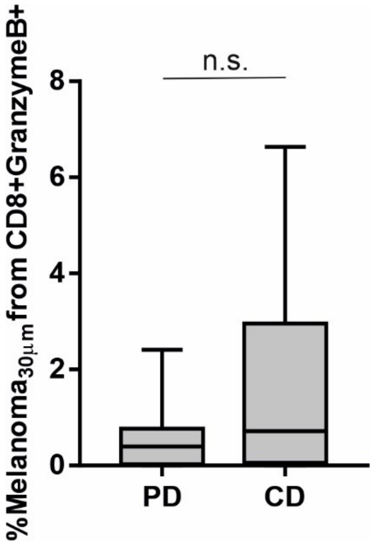

Supplementary Figure 2. Percentage of UM cells within a radius of 30 µm from CD8+Granzyme B+ T lymphocytes in PD and CD patients. Floating box extends from 25th to 75th percentiles, line through the box indicates median, and bars extend from the smallest to largest values. Non-parametric Mann-Whitney statistical analysis was performed across the two groups.

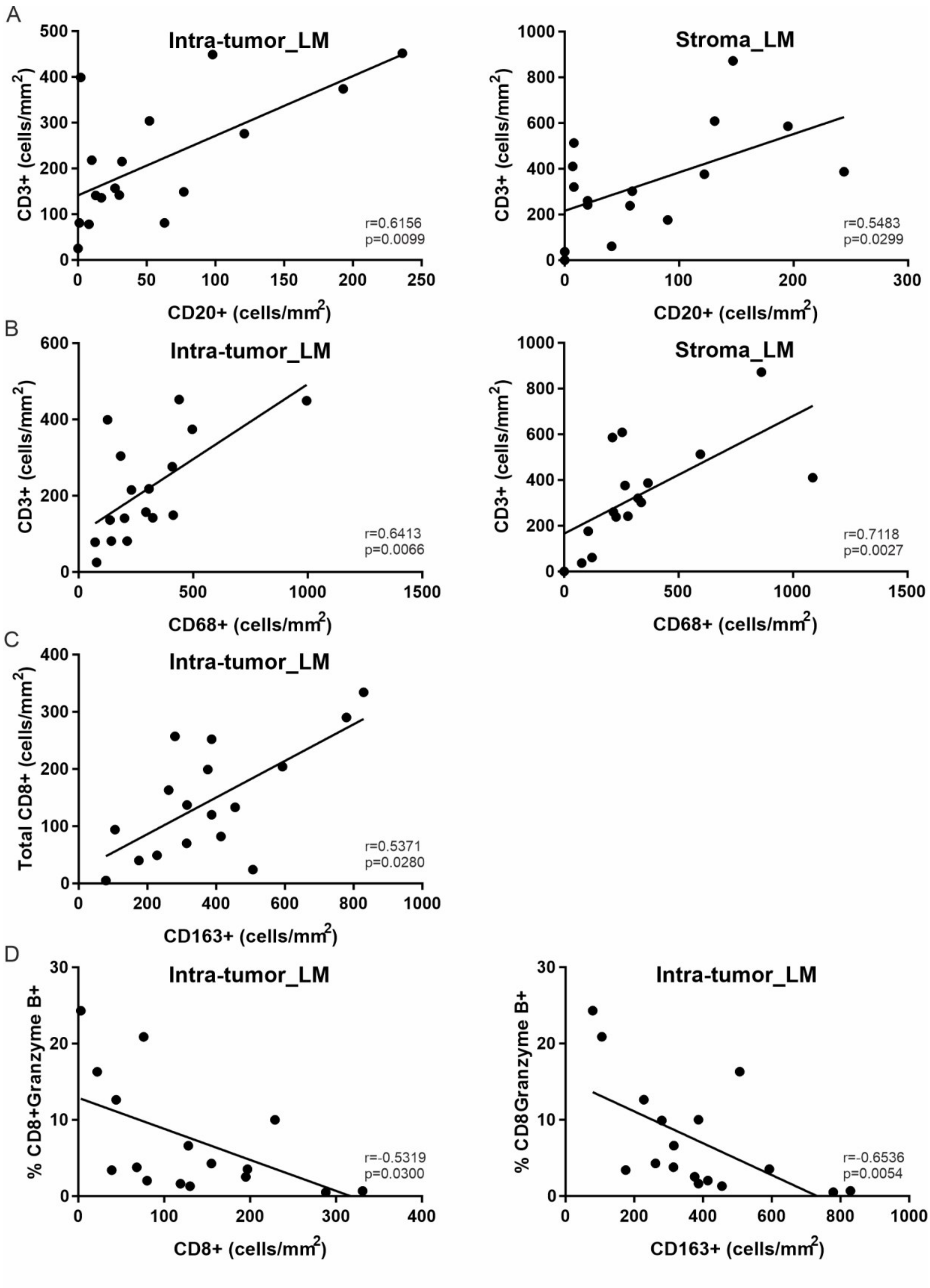

Supplementary Figure 3. Correlation analyses. Correlation between different immune cell infiltrates in LM within the tumor and in the stroma regions. Data are represented in a scatter plot with the best fit shown as solid line. The non-parametric Spearman's correlation coefficient (r) and p value were calculated for each graph.

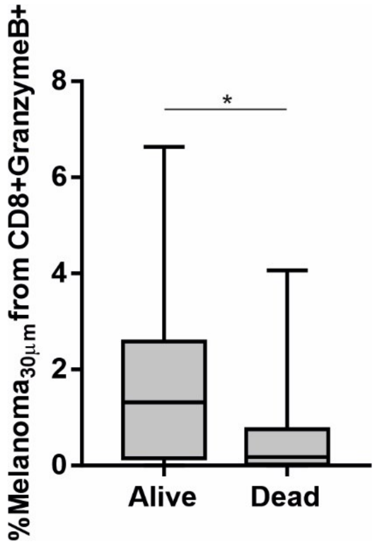

Supplementary Figure 4. Percentage of UM cells within a radius of 30 µm from CD8+Granzyme B+ T lymphocytes in patients alive and dead at the last follow-up time point. Floating box extends from 25th to 75th percentiles, line through the box indicates median, and bars extend from the smallest to largest values. Non-parametric Mann-Whitney statistical analysis was performed across the two groups, and significantly different data is represented by *($p<0.05$).

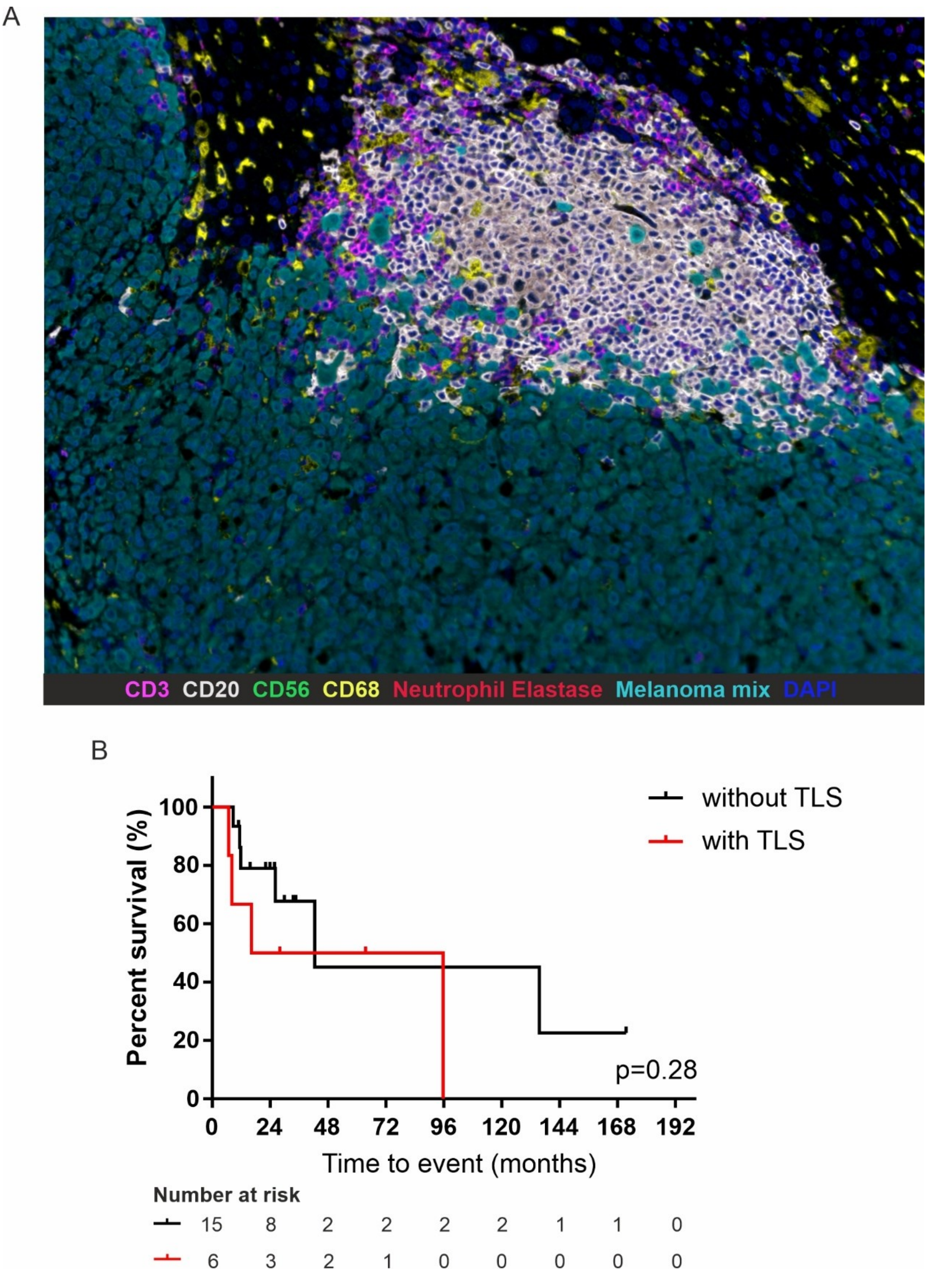

Supplementary Figure 5. The presence of TLS did not correlate with metastatic UM patient's prognosis. A) Representative 7-color mIHC image of a TLS found in a metastatic UM sample. Markers and color code are indicated under the picture. Original magnification 20X. **B)** Kaplan-Meier curves for overall survival according to the presence or absence of TLS in the tumor microenvironment. Log-rank statistics were performed to determine significance, p value and the number of patients at risk for each time point are reported.

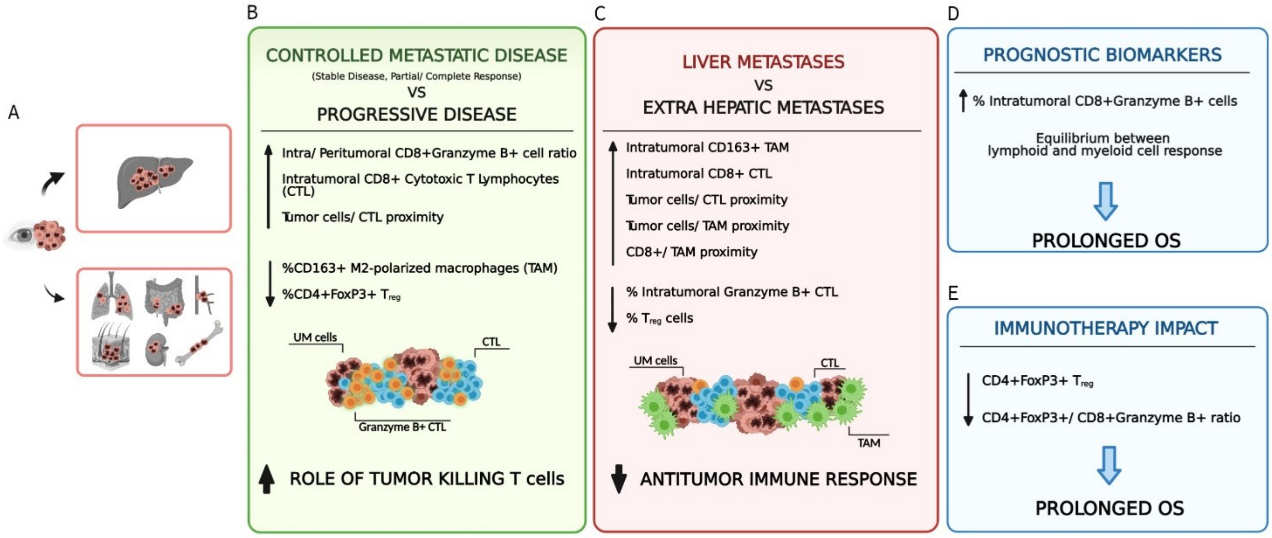

Supplementary Figure 6. A schematic cartoon illustrating the key findings of the manuscript.
